# Supplementary material for: Chromothripsis during telomere crisis is independent of NHEJ, and consistent with a replicative origin
Source: Genome Res. 2019 May;29(5):737–49. doi: 10.1101/gr.240705.118 (PMC6499312; doi:10.1101/gr.240705.118)
Supplement: Supplemental Material [file supp_gr.240705.118_Supplemental_file_1.zip › contigs/annotated_contigs/DB77/contig.2.DB77_length_333_mean_cov_5.40540540541.docx]

**DB77_length_333_mean_cov_5.40540540541**

```
ATCGTATGTAAAATTTAGTGATGTCACATGAATTTGCTTAAAGTATAGATTATTTTCATAATTCTAGGAAACCAGATATTAAGCACTAA
 >chr7:56804394-56804510 - E=2e-55
ACTTATTCTAGGAGTTTAGTTATTATA|CCAGCATGGTGAAACCCCATCTCTACTAAAAATACACAAATTAGCCAGGCATTGTGGTGTG
                             >chr7:56801948-56802165 - E=2e-119
TGCCTGTAATCCCAGCTACTTGGGAGGCTAAAGTAGGAGGTGGAGGTTGCAGTGAGCCGAGATCGTGCCACTGCACTGCAGCCTAGTCG

ACAGAATGAGACTCCGTCTGAAAAATATATATACATTTATTAAGTGAACTTTCCAAAAAAAGTGGAT
```
